# Hybrid Frozen Elephant Trunk for Single-Stage Kommerell Diverticulum and Type A Dissection Repair

**DOI:** 10.1016/j.atssr.2022.12.005

**Published:** 2022-12-17

**Authors:** Santiago Besa, Fadi Hage, Aashish Goela, Michael W.A. Chu

**Affiliations:** 1 Division of Cardiac Surgery, Department of Surgery, Western University, London, Ontario, Canada; 2 Department of Medical Imaging, Western University, London, Ontario, Canada

## Abstract

Treatment of Kommerell diverticulum is challenging, often involving multistage repair to achieve successful exclusion of the aneurysmal diverticulum, division of the vascular ring to release compression, and reconstruction of the aberrant subclavian artery to maintain arm perfusion. A well-planned, elective repair remains the optimal scenario; however, emergency presentation can be extremely challenging, particularly in acute aortic dissection. We describe an innovative, single-stage approach using a novel multibranched hybrid arch frozen elephant trunk prosthesis to achieve complete repair of a Kommerell diverticulum and aberrant right subclavian artery in a patient who presented with an acute type A aortic dissection.

Kommerell diverticulum (KD) is an aneurysmal dilation of the origin of an aberrant subclavian artery. It is most commonly associated with an aberrant right subclavian artery (ARSA) arising from a left-sided aortic arch (63%), which can result in dysphagia lusoria and has been reported to carry a high risk of rupture or dissection up to 53%.^1^ Most patients with KD can be treated with medical observation; however, when surgery is warranted, many options, including several open and endovascular techniques, have been employed. The hybrid arch frozen elephant trunk (FET) technique has revolutionized the treatment of complex aortic arch disease, allowing comprehensive arch reconstruction in a single-stage procedure. We present the case of a patient with a type A aortic dissection (TAAD) that was found to have an ARSA with the dissection flap originating from the KD who was successfully treated with a hybrid arch FET, repairing both his TAAD and KD in a single-stage operation.

A 71-year-old man presented with a severe hypertensive crisis and was found to have a TAAD. Computed tomography (CT) scan demonstrated an ascending aorta measuring 67 mm, arch of 45 mm with a KD and ARSA causing esophageal compression. The entry tear originated from the KD with retrograde extension (Figure 1). Echocardiography demonstrated mild to moderate aortic regurgitation and normal left ventricular function. Although several options were considered, a decision was made for urgent surgical reconstruction with a single-stage hybrid arch FET reconstruction using the multibranched Thoraflex hybrid prosthesis (Terumo) to treat the entire dissected aorta, to exclude the origin of the KD, to disconnect the vascular ring, and to revascularize the right subclavian artery.

**Figure 1:**
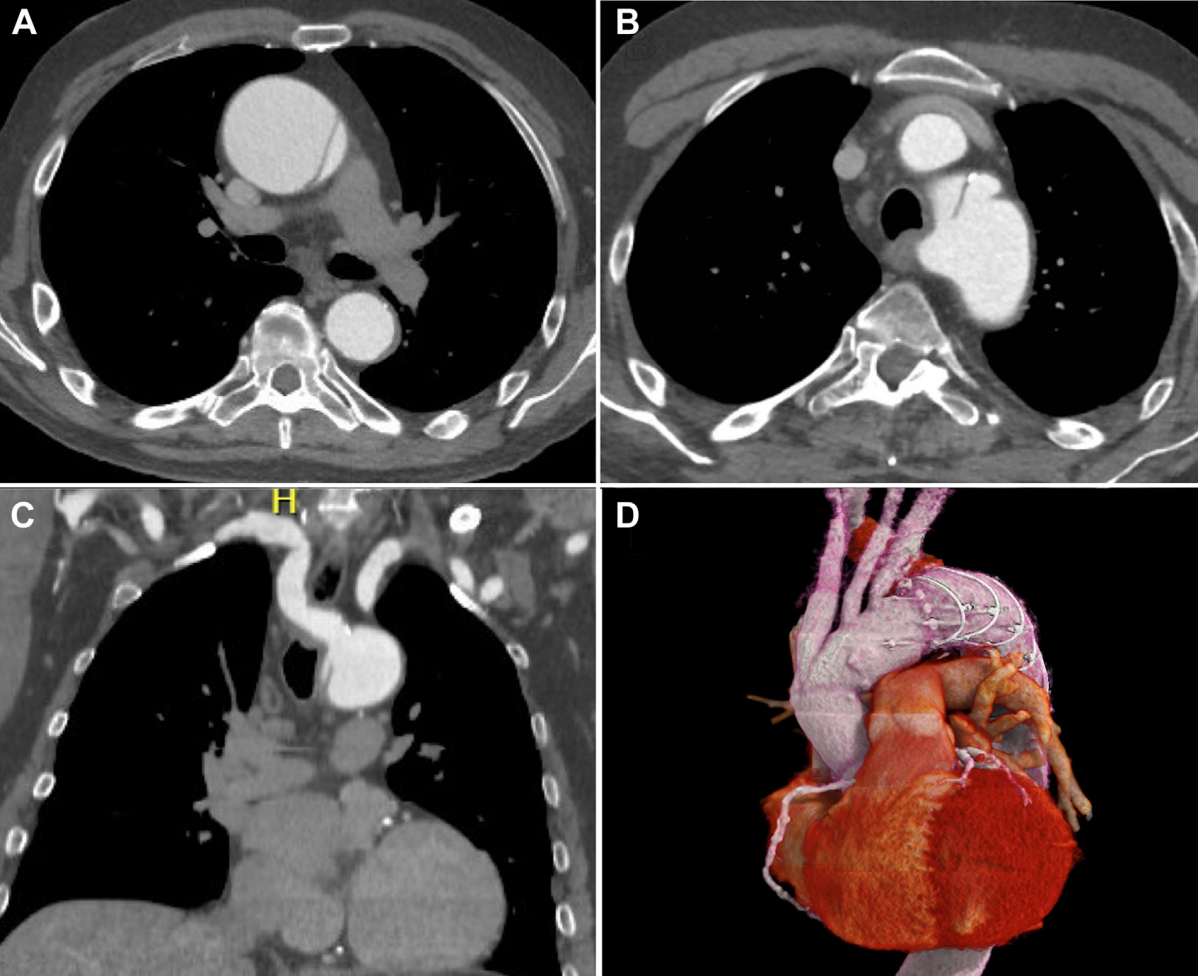
(A) Preoperative computed tomography (CT) scan showing an acute type A aortic dissection. (B) Preoperative CT scan showing the entry tear originating at the level of the Kommerell diverticulum. (C) Preoperative coronal CT reconstruction showing the origin and course of the aberrant right subclavian artery. (D) Postoperative 3-dimensional CT reconstruction showing the hybrid frozen elephant trunk and all its branches and the thrombosed Kommerell diverticulum.

A median sternotomy was performed. The ARSA was dissected out of the posterior mediastinum and controlled deep beside the trachea and esophagus. It was transected, and the proximal end was ligated near the base of the KD, thus disconnecting the vascular ring. The distal end was anastomosed to an 8-mm Dacron graft for future reconstruction, and the same was performed with the left subclavian artery. Both grafts were attached to separate arterial limbs of the heart-lung machine for perfusion. Cardiopulmonary bypass was initiated, and antegrade cerebral perfusion through a 15F cannula in the right carotid artery was employed. The aorta was cross-clamped, and the aortic root was found intact and nonaneurysmal. The aortic valve was resuspended, and a 28-mm Dacron graft was used to secure the sinotubular junction. When 25 °C was reached, circulatory arrest was initiated, maintaining perfusion through the right carotid artery and both subclavian arteries. Both carotid arteries were controlled, and the arch was transected at zone 2. A 30 × 40 × 100-mm Thoraflex was deployed over a stiff guide wire advanced from the femoral artery. The size and length of the prosthesis were selected for nominal sizing and to ensure at least 3 cm of overlap in the distal landing zone for safe coverage of the KD. The left carotid and subclavian branches were then anastomosed to reestablish bilateral antegrade cerebral perfusion. The proximal extent of the Thoraflex was anastomosed to the ascending aortic graft. Finally, the right carotid artery was anastomosed to the innominate limb of the Thoraflex, and the right subclavian artery graft was anastomosed in a Y fashion to the same limb (Figure 2). A Video summarizes the operative steps.

**Figure 2:**
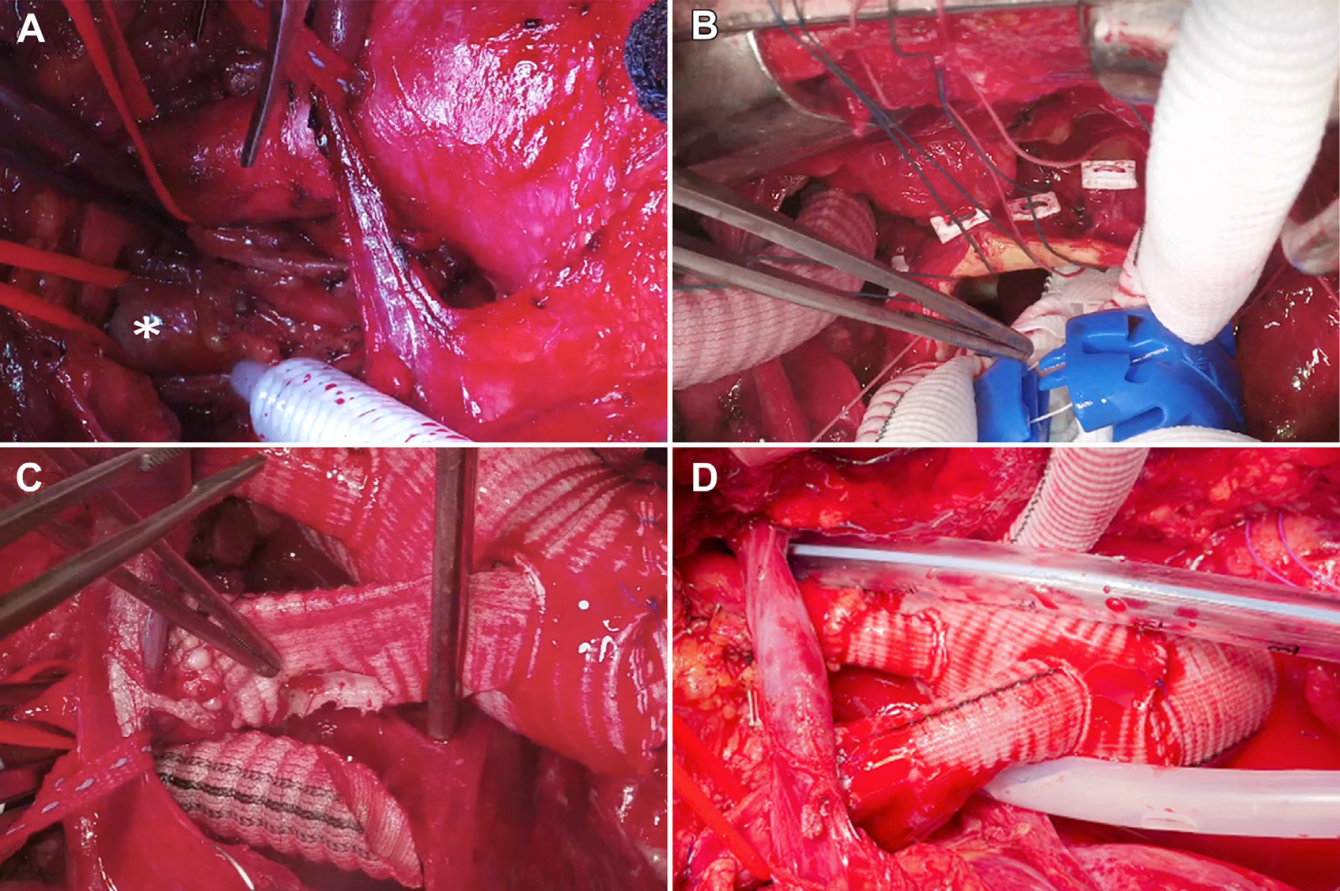
Intraoperative images. (A) The aberrant right subclavian artery, deep in the mediastinum (∗). (B) The deployment of the hybrid frozen elephant trunk prosthesis in zone 2. (C) Completed right carotid limb and preparation for anastomosis of the aberrant right subclavian artery graft to the right carotid limb. (D) Final result.

Surveillance CT scan demonstrated an intact 4-vessel aortic arch reconstruction with the FET well deployed into the descending thoracic aorta with complete exclusion of the KD (Figure 1D). The patient had transient delirium but was discharged home on postoperative day 15 without any further complications.

## Comment

The classic surgical repair consists of the resection of the KD, often requiring a descending thoracic aortic replacement and the division of the ARSA through a left thoracotomy plus a right carotid–to–subclavian artery bypass. Endovascular techniques obviate the need for a thoracotomy by excluding the aneurysmal KD and origin of the ARSA but leave the vascular ring intact without relief of any esophageal compression and remain at risk for endoleak complications. Many variations based on local expertise, anatomic variability, and patient preferences exist.^2^ Emergent presentation of the patient, such as in contained aortic rupture or TAAD, adds further complexity that can limit therapeutic options, depending on the expertise of the local aortic team and the need for simplified, single-stage repair.

The advent of hybrid arch devices allows single-stage treatment of extensive thoracic aortic disease. Moreover, the use of a specifically designed multibranched hybrid FET graft both simplifies the procedure and allows more flexibility in dealing with challenging aortic arch anatomy, such as in our patient with a KD and an ARSA. The unique design of the Thoraflex allowed us to replace the dissected ascending aorta and arch, to treat the entry tear at the level of the KD, to exclude the KD, and to revascularize the subclavian artery all at the same time through median sternotomy access alone. Other hybrid FET devices, such as the E-Vita Open Plus (Jotec) or the Cook hybrid (Cook Medical), could also have been used for the same purpose, but their lack of epiaortic branches would have required alternative reconstructive solutions. One of the caveats of FET is its higher risk of spinal cord injury, predominantly associated with longer stent grafts (>120 mm) and coverage below T8.^3^ This is of the upmost importance in this case, in which the stent graft length had to be selected to ensure complete exclusion and thrombosis of the KD. In the presented case, preoperative planning with CT scan predicted that a 100-mm stent graft would provide sufficient distal landing zone coverage while excluding the origin of the KD. The FET served to provide an additional level of safety to seal the KD, in addition to the posterior mediastinal suture ligation. This case also highlights the utility of multiple cannulation sites for perfusion that may improve perfusion safety in TAAD and enhance cooling and rewarming times.^4^

This case demonstrates the utility of novel, multibranched hybrid FET in treating complex TAAD and its many advantages in complex aortic disease. This is important in emergent presentations, when avoiding second-stage procedures may improve results and be associated with a lower risk of interstage death.^5^
